# Social and auditory experience shapes forebrain responsiveness in zebra finches before the sensitive period of vocal learning

**DOI:** 10.1242/jeb.247956

**Published:** 2024-10-25

**Authors:** Katie M. Schroeder, Luke Remage-Healey

**Affiliations:** ^1^ Graduate Program in Organismic & Evolutionary Biology, University of Massachusetts Amherst, Amherst, MA 01003, USA; ^2^ Center for Neuroendocrine Studies, University of Massachusetts Amherst, Amherst, MA 01003, USA

**Keywords:** Auditory perception, Critical period, Electrophysiology, Plasticity, Songbird, Species recognition

## Abstract

Early-life experiences with signals used in communication are instrumental in shaping an animal's social interactions. In songbirds, which use vocalizations for guiding social interactions and mate choice, recent studies show that sensory effects on development occur earlier than previously expected, even in embryos and nestlings. Here, we explored the neural dynamics underlying experience-dependent song categorization in young birds prior to the traditionally studied sensitive period of vocal learning that begins around 3 weeks post-hatch. We raised zebra finches either with their biological parents, cross-fostered by Bengalese finches beginning at embryonic day 9, or with only the non-singing mother from 2 days post-hatch. Then, 1–5 days after fledging, we conducted behavioral experiments and extracellular recordings in the auditory forebrain to test responses to zebra finch and Bengalese finch songs. Auditory forebrain neurons in cross-fostered and isolated birds showed increases in firing rate and decreases in responsiveness and selectivity. In cross-fostered birds, decreases in responsiveness and selectivity relative to white noise were specific to conspecific song stimuli, which paralleled behavioral attentiveness to conspecific songs in those same birds. This study shows that auditory and social experience can already impact song ‘type’ processing in the brains of nestlings, and that brain changes at this age can portend the effects of natal experience in adults.

## Teaser

**Summary:** Tuning of neural circuits for species-specific song recognition can be altered in very young songbirds before the sensitive period of vocal production learning.

## INTRODUCTION

Early-life experiences with signals used in communication can be instrumental in shaping an animal's social interactions. From graylag geese that imprint on the first moving object they encounter after hatching (Lorenz, 1937), to human infants' increased proficiency for discrimination of native over non-native language phonetics within the first year of life (Kuhl et al., 2006), primary social interactions leave a strong impression. Across multiple taxa, early experiences can influence later signal discrimination (Maye et al., 2002; Pardo-Sanchez et al., 2022), signal production proficiency (Johnson and Newport, 1989) and mate preference (Verzijden and ten Cate, 2007). Only recently, however, has the field of developmental science recognized that altricial songbirds, which hatch naked with closed eyes, can engage in prenatal and early postnatal learning.

It was previously presumed that auditory experience during the nestling phase does not affect songbirds because they are not fully developed at hatching (Dooling and Searcy, 1980; Marler and Peters, 1989). This position was strengthened by studies showing that zebra finches (*Taeniopygia guttata*) do not memorize and ultimately reproduce songs they hear before a ‘sensitive period’ opens at approximately 3 or 4 weeks of age (Braaten, 2010; Eales, 1985; Roper and Zann, 2006). Taken together, those studies found no evidence of vocal learning in songbirds younger than ∼22–25 days old. Yet several recent studies in multiple songbird species provide evidence for learning and experiential effects in both embryos and nestlings (Colombelli-Négrel et al., 2012; Hudson et al., 2020; Langmore et al., 2008; Madden and Davies, 2006; Mariette and Buchanan, 2016; Schroeder and Podos, 2023; but see Wheatcroft and Qvarnström, 2017). For instance, swamp sparrow (*Melospiza georgiana*) nestlings less than 10 days of age were found to discriminate between subspecies dialects after experience with song types from more than one subspecies (Schroeder and Podos, 2023). These findings predict that a trace of early developmental experience is present already in nestling songbird brains. However, neurophysiological evidence is lacking for sensory learning and plasticity in songbirds before the onset of the traditional vocal learning period, and assessment of neural dynamics underlying early learning would push the field forward.

Here, we focused on learning of species-level song categories. Songbirds are thought to have an approximate conspecific template at or before hatching that guides preferences for learning and attending to songs with characteristic, species-specific acoustic features (Marler, 1970; Soha, 2017). From egg to adult, there is overwhelming evidence for conspecific song selectivity (Colombelli-Négrel et al., 2014; Marler and Peters, 1977; Nelson and Marler, 1993), particularly in auditory forebrain regions Field L, NCM and CMM (reviewed in Louder et al., 2019; Woolley, 2017). For these brain regions in adults, same-species song preferences, neural tuning and stimulus selectivity can be altered by experience (reviewed in Woolley, 2017), and even the strong conspecific selectivity can be partially overridden in some cases: zebra finches raised by Bengalese finches eventually produce Bengalese syllable types, duration and number, but retain zebra finch-like syntax and gap duration (Araki et al., 2016; Clayton, 1989). It remains unknown when changes in neural responses and tutor preference begin or how they manifest in the brain during development. Recent evidence suggests that experience may allow changes in song categorization during the nestling phase (Schroeder and Podos, 2023), and that the auditory system of young songbirds is already adult-like well before the motor learning pathway matures (Schroeder and Remage-Healey, 2021). Thus, experience during the earliest phases of development could affect species-level recognition and neural processing well before the sensitive period of vocal learning.

**Fig. 1.**
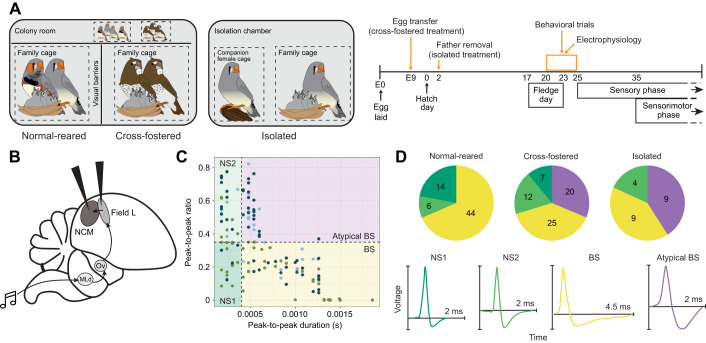
**We recorded single units from the auditory forebrain in response to songs of different species in birds raised in different social environments (normal-reared=8, cross-fostered=6, isolated=3 birds).** (A) Experimental setup and timeline of the three social treatments. Normal-reared and cross-fostered birds were raised in family cages within a colony room containing other adult zebra and Bengalese finches, but with visual access only to their family members. Isolated birds were raised by only their biological mother in an isolation chamber containing an unrelated female housed in a separate cage. (B) Songbird brain diagram indicating direction of information in the auditory circuit. (C) Waveform shape characteristics of all 150 NCM single units (group sample sizes in D) colored by social treatment group, labeled with their unit type: narrow-spiking 1 (NS1), narrow-spiking 2 (NS2), broad-spiking (BS) and atypical broad-spiking (Atypical BS). Unit waveforms are separated above and below 0.4 ms and 0.35 peak-to-peak ratio. Previous studies in adults (Bottjer et al., 2019; Macedo-Lima et al., 2021) did not find any units in the upper right quadrant (here, Atypical BS). We have plotted these units as a separate group in Fig. 2A, but they are lumped with BS units for all statistical analyses. (D) Distribution of unit types within each treatment group, and unit type exemplar unit shapes.

In this study, we tested the hypothesis that song preference behaviors and response properties of auditory forebrain neurons that contribute to those behaviors are altered by social and auditory experience between hatching and the opening of the classic song-learning sensitive period (i.e. experience in the first 25 days post-hatch). To do this, we raised zebra finches in three different treatments (Fig. 1A): with both biological parents (‘normal-reared’), cross-fostered by Bengalese finches (*Lonchura striata domestica*) (‘cross-fostered’) or with only their non-singing mother (‘isolated’). One to five days after birds fledged from the nest (ages 20–25 days post-hatch), we measured fledgling behavior and the electrophysiological responses, in Field L and NCM, to adult songs of zebra and Bengalese finches.

**Fig. 2.**
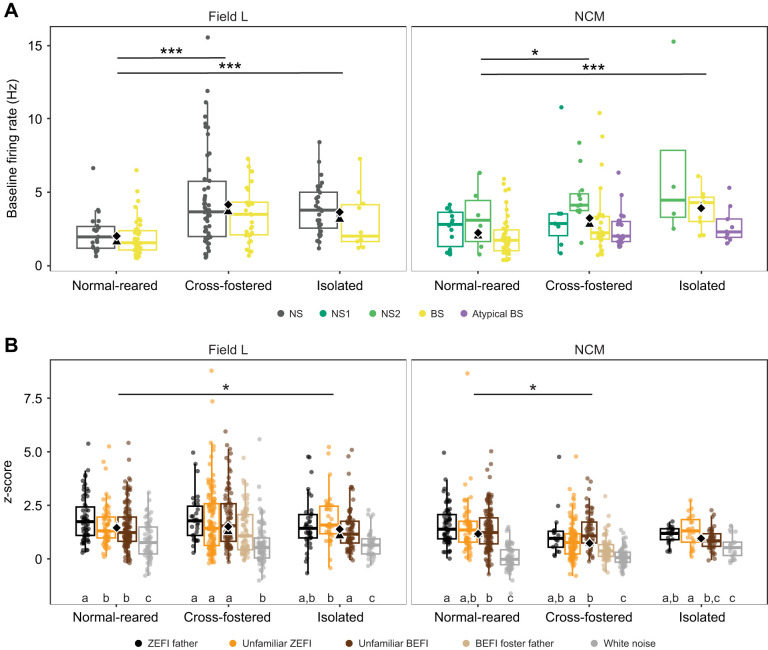
**Abnormal social rearing environments led to increased firing activity, decreases in responsiveness and decreases in firing consistency in response to zebra finch songs in the auditory forebrain.** (A) Baseline (spontaneous) firing rates as a function of brain region (panels), treatment and unit type (point color). Atypical BS units are plotted separately here for visual comparison but were lumped with BS units for statistical analyses. Units from cross-fostered and isolated birds had higher firing rates than units from normal-reared birds in Field L (GLMMs with Tukey correction all *P*<0.0001) and NCM (GLMM with Tukey correction, normal–isolated *P*<0.001, normal–cross-fostered *P*=0.03). (B) *Z*-score responses to each stimulus category (point colors) as a function of brain region (panels) and treatment. Each point represents a unit–stimulus combination. Each stimulus was presented to each unit (see sample sizes in Table 1) 15–20 times. *Z*-scores for units from cross-fostered birds in NCM were higher in response to uBEFI as compared with uZEFI songs (LMM with Tukey correction, *t*_655_=4.96, *P*<0.0001). In both A and B, letters below boxplots show contrasts within treatment groups with *P*>0.05. Lines above each plot show contrasts between treatment group means where ***P>0.001; **P>0.01; *P>0.05.

**Table 1.**
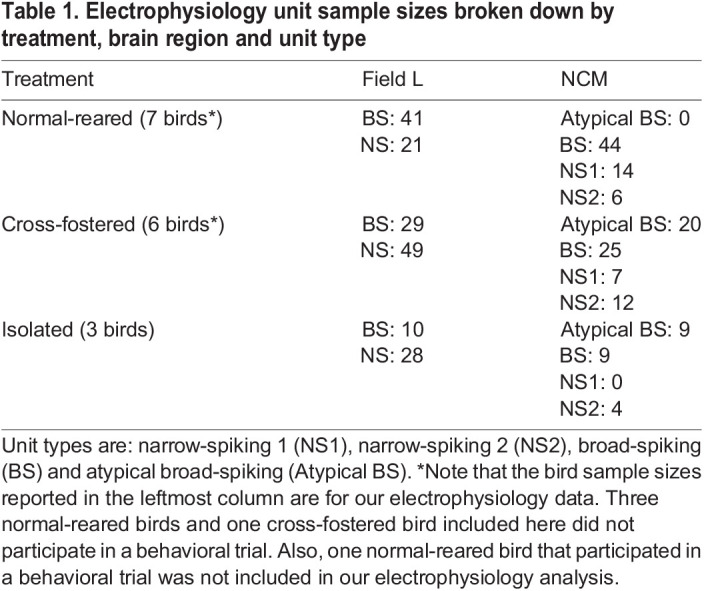
Electrophysiology unit sample sizes broken down by treatment, brain region and unit type

If the learning and refinement of preference for conspecific song occurs together with the sensitive period for song memorization at 3–4 weeks old, then our cross-fostered treatment should have no effect on subsequent auditory behaviors and neuronal responses. If, however, species preferences can be learned before song models are memorized, during the first 3 weeks of life, then we would predict three main outcomes. First, and most generally, we predict that auditory forebrain neurons in birds from different rearing environments will show different response properties. Second, we predict that cross-fostered birds will show, compared with normal-reared birds, a weaker behavioral preference for, and neural responsiveness/selectivity to, conspecific songs compared with heterospecific songs (i.e. from Bengalese finches) (Hauber et al., 2013; Logerot et al., 2020). Third, following studies suggesting that nestlings do not yet memorize individual song exemplars (Braaten, 2010; Eales, 1985; Roper and Zann, 2006), we predict that auditory forebrain neurons in recent fledglings will not respond differently to songs from familiar (father) and unfamiliar individuals. Therefore, this study sets out to connect experience-dependent changes in behavioral song preferences with observed changes in neural activity patterns at an age previously unexplored in songbirds.

## MATERIALS AND METHODS

### Subjects and early experience timeline

The experiments were conducted with the captive colonies of zebra finches [*Taeniopygia guttata* (Vieillot 1817)] and Bengalese finches (*Lonchura striata domestica* Linnaeus 1758; also called society finches) at the University of Massachusetts in Amherst, MA, USA (housed on a 14 h:10 h light:dark cycle). To test the effect of early auditory and social experience, we raised zebra finches in one of three experimental setups: normal-reared (control), cross-fostered or isolated (Fig. 1A). For all treatment groups, we limited clutches to a maximum of three chicks per nest to avoid neural and behavioral effects of developmental stress (Sewall et al., 2018).

Normal-reared juveniles (*N*=8) were raised by their biological zebra finch parents. Five zebra finch pairs were each used once to produce and/or raise a single clutch. Normal-reared juveniles were raised in single-family cages (i.e. mother, father and offspring) with visual access only to their parents and siblings but auditory access to adult zebra finches and Bengalese finches housed in the same room. This ensured that the song of the father would be most salient as a tutor model (Chaiken et al., 1993; Chen et al., 2016; Eales, 1989; Varkevisser et al., 2022), but also that songs of both species would not be entirely novel to the young birds.

For the cross-fostered treatment, juveniles (*N*=6) were raised by a pair of Bengalese finches. We transferred zebra finch eggs to a Bengalese finch nest before incubation day E9 (Fig. 1A). Moving eggs before this time point was intended to safeguard against effects of prior auditory experience with biological fathers because: (1) behavioral responses to auditory stimuli begin by E16 in precocial chickens (Jackson and Rubel, 1978), which is approximately the same developmental time point as E9 in zebra finches; and (2) E9 is also the day before zebra finch parents begin incubation calls shown to affect subsequent calling and growth rates in the nestlings (Mariette and Buchanan, 2016). Prior to egg transfer, the Bengalese finch pair was either already incubating eggs of their own or had been induced to incubate fake eggs. All Bengalese eggs or fake eggs were removed when the zebra finch eggs were transferred. One male, DB22P31, and two different partner Bengalese finches (one of whom was confirmed ZW female) were used to raise all three cross-fostered clutches.

The surrounding auditory environment of the normal-reared and cross-fostered treatment groups was the same; only the immediate social and auditory environment differed. The cross-fostered juveniles were raised in family cages in the same room as the normal-reared juveniles and were also visually restricted to individuals in their family cage. To ensure that chicks in both treatment groups gained baseline levels of experience with their social fathers' (i.e. the bird raising the chick) songs, we placed speakers immediately above the nest boxes to broadcast playback of the social father's (biological or foster) song for 2 h at a rate of 1 song min^−1^ each morning during the nestling period. This is not unnatural experience given that wild zebra finch males sing during the brooding period at a rate of approximately 1.4 phrases per 5 min, with approximately 70% of undirected song occurring within 1 m of the nest (Dunn and Zann, 2010). We ended this song playback on the day after the first fledgling left the nest box.

For the isolated treatment group (*N*=3), juveniles were raised in a sound attenuation chamber (Eckel Acoustics) with a single zebra finch mother and an unrelated companion female in a separate cage. These juveniles heard social vocalizations as well as very few male songs. For this single clutch, the father was removed 2 days after the chicks hatched (Fig. 1A). Several attempts were made to remove the male on day E9 to achieve full isolation from male song, but each time we did this the single females stopped incubating. We used Sound Analysis Pro (Tchernichovski et al., 2000) and an omnidirectional microphone (Countryman) to record the number of songs the male sang between day E9 and his removal. The father sang only six song bouts between E9 and E13 (hatch day), including only two song bouts between the first hatched chick and his removal from the isolation chamber, all less than or equal to 4 s in duration.

### Behavioral trials

We conducted behavioral trials 1–5 days post-fledge (age range=20–25 days post-hatch, age average=22.5 days post-hatch). At this age, zebra finches do not sing yet and are still fully dependent on parents. Plumage is not yet sexually dimorphic, and we did not attempt to determine the sex of the individuals in this experiment. At this age, sex differences in auditory processing are not pronounced, and conspecific discrimination is presumed to be important in both sexes, so we did not predict significantly different song responses between males and females. During trials, we placed each fledgling individually in a sound attenuation chamber. After 5 min of silent acclimation time, we played 1 min of zebra finch or Bengalese finch song, then 1 min of silence, then 1 min of the other species' song. Songs were broadcast at a maximum level of 52 dB (A-weighting at 20 cm from source) at a rate of 1 song every 10 s. The order of song presentation was chosen pseudorandomly for each individual and the stimuli were chosen randomly from a set of four zebra finch songs and four Bengalese finch songs. Subjects were videoed, and later scored for attentiveness as measured by the number of head turns, head tilts, neck stretches and gaping. We counted these behaviors during song playback as well as the silent minute preceding each playback period to estimate the change in behaviors over baseline. We interpreted these behaviors as visual search for the singer of the stimulus (Fernández-Juricic, 2012), with higher activity representing higher attentiveness and interest. Our young birds performed only a few, subtle movements during the behavioral trials, which precluded our use of the absence of behavior as a measure of attentiveness, as in other studies on older juveniles (e.g. Chen et al., 2016).

### Surgery

On the day of the behavioral trial (or on the day after for two individuals), we performed extracellular electrophysiological recordings *in vivo*. Because these recent fledglings were still dependent on their parents for food and thus could not be isolated, we performed a headpost and craniotomy surgery on the day of recording. Birds were fasted for 30 min prior to anesthesia, usually including time spent in behavioral trials. At 15 min prior to anesthesia, we weighed the birds and gave an oral dose of 15 μl meloxicam (∼0.3 mg kg^−1^). Birds were anesthetized with 2.5–3% isoflurane in 2 l min^−1^ O_2_, wrapped in a cloth blanket, and fixed to a custom stereotaxic apparatus (Herb Adams Engineering) at a head angle of 45 deg. During surgery, birds were maintained on 1% isoflurane in 2 l min^−1^ O_2_ and at a body temperature of 36°C using a heating pad and DC temperature controller (﻿FHC NeuroCraft). Following a subcutaneous lidocaine (2% in ethanol) injection to the scalp, we exposed the skull and located the midsagittal sinus bifurcation to use as a zero-point reference. We marked the skull to indicate left and right NCM at 1.1 mm rostral and 0.7 mm lateral to the reference point and left and right Field L at 1.6 mm rostral and 1.2 mm lateral. Small craniotomies were made at these locations by removing skull tissue – which is very thin and flexible at this age – and carefully resecting the dura to expose the brain surface. We then fixed a metal head post at the base of the bird's beak with dental cement to stabilize the head during recordings. Birds were allowed at least 10 min (average=23 min) to recover from anesthesia before being transferred to the recording booth, although most birds were awake within 2 min.

### Dual-site extracellular electrophysiology and song playback

Immediately following surgery and recovery, we performed *in vivo* awake-restrained extracellular recordings as in previous studies (Miller-Sims and Bottjer, 2014; Schneider and Woolley, 2013; Schroeder and Remage-Healey, 2021; Vahaba et al., 2020). Birds were restrained in a custom body-wrap and head-fixed to a stereotaxic head-post holder (Herb Adams Engineering) at a head angle of 45 deg on an air table (TMC) in an anechoic booth (Industrial Acoustics). Single 500 kΩ tungsten electrodes (A-M Systems) were lowered independently and simultaneously into NCM (caudomedial nidopallium) and Field L in the same hemisphere (Fig. 1B). We pseudorandomly began with the right or left hemisphere for each bird, attempting to obtain an even number of recordings from both hemispheres across birds. Between 0.92 and 1.88 mm ventral to the brain surface for NCM and 0.76 and 1.79 mm ventral to the brain surface for Field L, we located auditory-responsive sites and characteristic background firing patterns using pure tones and songs that were not part of the test set. Recording sites from a single electrode penetration were separated by at least 50 μm. If a second penetration was made in the same region (44% of all bird–hemisphere–region combinations), we avoided recording sites at depths recorded during the first penetration. For each bird, we recorded from an average of eight sites in each brain region across both hemispheres. Recordings were amplified, bandpass filtered (300 to 10,000 Hz; A-M Systems) and digitized at 20 kHz (Micro 1401, Spike2 software; Cambridge Electronic Design).

From a pool of 22 songs, we created a unique set of five sound stimuli for each bird. Normal-reared and isolated birds heard their biological father's song, one unfamiliar zebra finch song, two unfamiliar Bengalese finch songs and white noise. Cross-fostered birds heard their biological father's song, their Bengalese foster father's song, one unfamiliar zebra finch song (uZEFI), one unfamiliar Bengalese finch song (uBEFI) and white noise. No birds heard the same song stimulus during both their behavioral trial and the extracellular recording. Each unfamiliar song stimulus was only included in a stimulus set for an average of 2.4 birds (maximum=5 birds). The stimuli ranged from 1.2 to 2.87 s each in duration and were calibrated to a peak amplitude of 52 dB at 1 m, with the bird's head positioned 45 cm from the speaker. At each recording site, one trial consisted of 15 repetitions (or 20 repetitions for two individuals) of each of the five stimuli in random order with an interstimulus interval of 10±2 s, thus each trial lasted approximately 15 min.

Recordings lasted a maximum of 4 h. Following the recording, birds were killed via rapid decapitation. Brains were extracted and drop-fixed in 20% sucrose in 10% formalin, then stored at −80° C for later verification of electrode placements. All experimental procedures were approved by the Institutional Care and Use Committee of the University of Massachusetts Amherst (protocol no. 3613).

### Single-unit spike sorting and analysis

We processed recordings in Spike2 software (version 7.04). We identified single units, distinctly shaped action potentials presumed to represent a single neuron, by first setting a unique threshold for each recording site to two times the noise band (background fluctuations in voltage not attributed to neuronal action potentials) or 1.5 times the noise band for some Field L recordings with lower signal-to-noise ratios. We sorted spikes (i.e. action potentials) that crossed this threshold using a principal components plot based on waveform shape and retained well-isolated units as determined through *K*-means analysis (J3 mean±s.e.m.=5.03±0.17; where J3>2 indicates well-sorted clusters; Wheeler, 1998). None of our single units had an interspike interval <1 ms. We further excluded units from analysis that we classified as non-auditory because they did not respond significantly (*P*<0.05 change from baseline to stimulus-evoked firing rates) to at least one of the five stimuli, as determined using paired Wilcoxon signed-rank tests. The percentages of units excluded for being non-auditory were as follows: normal-reared, Field L=16%, NCM=17%; cross-fostered, Field L=12%, NCM=26%; and isolated, Field L=5%, NCM=0%.

For all units retained in the analysis (*N*=126 normal-reared, *N*=142 cross-fostered, *N*=60 isolated; Table 1), we measured the following waveform properties from an average rendering of all spikes for each unit in Spike2: peak-to-trough duration, peak amplitude and trough amplitude. In NCM, these waveform properties provide insight into how individual units are involved in circuits, either as excitatory projection neurons (long/broad in duration) or inhibitory interneurons (short/narrow in duration; Spool et al., 2021). NCM units were separated into broad-spiking (BS), narrow-spiking 1 (NS1) and narrow-spiking 2 (NS2) units as in previous studies (Bottjer et al., 2019; Macedo-Lima et al., 2021), using as separation points a peak-to-trough duration of 0.4 ms and a peak-to-trough amplitude ratio of 0.35 (Fig. 1C,D). We found a unique subset of units in NCM of cross-fostered and isolated birds, but not in normal-reared birds, with a previously undocumented waveform shape. These units showed a combination of high peak-to-peak ratio, indicating high symmetry and long duration. Based on similar response profiles, we lumped these units with BS units for all statistical analysis but have plotted them separately in Fig. 2A as ‘Atypical BS’ units for visual comparison with other unit types. Field L studies typically do not distinguish unit types, but we found putative broad and narrow units in our dataset, which we separated at 0.4 ms, similar to previous studies (Calabrese and Woolley, 2015; Fig. S1). We additionally considered a cut-off of 0.75 ms, given the lack of unit density at this point on the *x*-axis in Fig. 1C. However, only two minor differences emerged in our conclusions when using the 0.75 ms cut-off versus the 0.4 ms cut-off: (1) baseline firing rates of units from normal-reared and cross-fostered birds were no longer different, and (2) accuracy of units from cross-fostered birds were no longer different for uBEFI and uZEFI stimuli. Because these differences would not significantly alter our main conclusions, we decided to report the results with the conventional 0.4 ms cut-off previously used in the literature.

For all units, we calculated several response metrics using Python and/or R (https://www.r-project.org/). Baseline firing rate is the number of spikes in the 1 s preceding each stimulus onset. We calculated a *z*-score to represent responsiveness using the following formula:
(1)

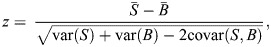
where *S* is the stimulus-evoked firing rate and *B* is the baseline firing rate. Firing rates and *z*-scores presented in this paper are averages of a unit's response over all presentations of a given stimulus, except in a few circumstances where an individual presentation trial was excluded owing to overlap with a movement artifact (range 0–18 out of 75 presentations removed from a single trial). We also calculated the proportion of all units that responded significantly over baseline firing rate (Wilcoxon signed-rank tests as above) to each stimulus.

We analyzed *d*′ as a metric of selectivity following methods in previous studies (Green and Swets, 1966; Mooney et al., 2001; Moseley et al., 2017; Remage-Healey and Joshi, 2012; Vahaba et al., 2020)*.* We followed the formula:
(2)

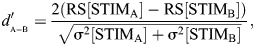
where STIM_A_ is the stimulus of interest, STIM_B_ is the comparison stimulus (white noise or unfamiliar Bengalese finch songs as indicated), RS is the response strength calculated by stimulus-evoked firing rate minus baseline firing rate, and σ is the RS variance.

To assess the ability of our fledglings to consistently encode within-song acoustic features of zebra finch and Bengalese finch songs, we used a custom pattern classifier in Python (Schroeder and Remage-Healey, 2021; Vahaba et al., 2017). This classifier assesses the variability of spike timing patterns across multiple presentations of the same stimulus. This metric, normalized *R*_corr_ accuracy, ranges from 0 to 1, with higher values indicating that the spike timing patterns better predict the stimulus. See further details in the Supplementary Materials and Methods.

Additional calculations and analyses regarding temporal firing properties and unit population-level assessment of responsiveness and selectivity can be found in the Supplementary Materials and Methods.

### Statistical analysis

We tested the hypothesis that early social environment affects fledgling behavioral and auditory forebrain responses to species-specific songs. All statistical analyses were performed in R. For analysis of our behavioral data, in recognizing the small sample size, we used a separate paired Wilcoxon signed-rank test for each treatment to compare behavior counts between the unfamiliar zebra finch playback period and the unfamiliar Bengalese finch playback period. These tests were performed using standardized behavioral count, which is the number of behaviors observed during the 1 min of song playback minus the number of behaviors observed during the 1 min of silence prior to playback.

For analysis of our electrophysiological data, we created a separate GLMM to model each response variable in each brain region. The Field L and NCM models for the same response variable (e.g. firing rate) always used the same distribution function but differed in parameter specification in a few circumstances when necessary to improve model fit and diagnostics. All models included a treatment×stimulus type interaction term and a unit type fixed effect, with the exception of the model for baseline firing rate, which did not include stimulus type. In NCM models, unit type was separated into broad-spiking (BS), narrow-spiking 1 (NS1) and narrow-spiking 2 (NS2) types, while Field L was only separated into BS and NS. To avoid pseudoreplication, we did not include the ‘foster father’ stimulus in statistical analysis because we only used a single foster father and his song for all cross-fostered birds (although it is included in all figures for qualitative visual comparison). Bird mass, hemisphere, age and days post-fledging on recording day were all considered as both fixed or random effects and were retained as one or the other if it improved the model fit (as determined using AIC) and diagnostics. Unit ID, clutch ID and bird ID were considered as random effects for all models and were retained if the variance for these variables was >0.01. In the end, clutch ID was not retained in any final model, bird ID was retained for half of the NCM models but no Field L model, and unit ID was retained in all final models. Model diagnostics and assumptions were checked using the DHARMa package (https://CRAN.R-project.org/package=DHARMa). *Post hoc* tests were conducted with the emmeans package (https://CRAN.R-project.org/package=emmeans) using the Tukey method to correct *P*-values for multiple tests. Mean values reported in the Results section represent estimated marginal means±s.e.m. calculated from models back-transformed to the response scale.

We created all models initially with the full dataset (total *N*=328) and subsequently without a subset of units that were sorted with less confidence (conservative *N*=213). After removal of those units, results obtained closely matched the results with the full dataset and so all units were retained in the final models.

For the *d*′ metrics, we also performed a cumulative distribution function (CDF) analysis. To compare these two metrics across treatment, stimulus type and region, we used the Levene's test in the ‘stats’ package of base R to analyze the variance, which is reflective of the slope of the cumulative distribution. Pairwise comparisons were again performed with a Tukey correction for multiple tests. Detailed statistical output from all models and analyses can be accessed on our GitHub repository (Schroeder, 2024).

## RESULTS

### Prediction 1: nestling experience shapes auditory forebrain physiology

All three treatment groups showed robust song-evoked responses in auditory forebrain (Fig. S2), but overall, we found that when social and auditory experience with conspecific adults was reduced, electrophysiological responses to auditory stimuli were altered, with changes generally more pronounced in NCM than in Field L. The first major difference was the discovery of a unique subset of NCM units (‘units’ are assumed to be single neurons) whose action potentials had long durations and high peak to trough symmetries, which were only seen in cross-fostered and isolated birds (Fig. 1D). Additionally, we found that isolated unit baseline firing rates were 1.9× higher than in normal-reared units in Field L (estimate±s.e.=−0.66±0.13, *z*=−5.11, *P*<0.0001) and 2.1× higher in NCM (estimate=−0.71±0.16, *z*=−4.49, *P*<0.0001), while cross-fostered unit baseline firing rates were 2.3× higher in Field L (estimate=−0.82±0.11, z=−7.23, *P*<0.0001) and 1.4× higher in NCM when compared with units from normal-reared birds (estimate=−0.35±0.14, *z*=−2.52, *P*<0.03; Fig. 2A).

Auditory single units from cross-fostered birds showed reduced responsiveness (*z*-score) to all stimuli combined as compared with units from normal-reared birds in NCM but not Field L (normal-reared NCM mean±s.e.m.=1.17±0.11, cross-fostered NCM mean=0.74±0.12, estimate±s.e.=0.42±0.16, *t*=2.70, *P*=0.02; Fig. 2B). This pattern was also notable when considering the proportion of auditory units that responded to a given stimulus (probability of responding, NCM cross-fostered=0.62, NCM normal-reared=0.81, contrast *P*<0.01; Fig. S3B).

Song selectivity, measured by *d*′ calculated with white noise as a reference (*d*′_WhiteNoise_), was reduced by more than half in isolated birds, and cross-fostered birds to a lesser extent, in NCM but not Field L (NCM statistics: normal-reared mean=2.2±0.26, cross-fostered mean=1.50±0.26; isolated mean=0.98±0.40, contrast normal-reared versus cross-fostered estimate=0.74±0.36, *t*=2.08, *P*=0.09; contrast normal-reared versus isolated estimate=1.26±0.46, *t*=2.73, *P*=0.02; Fig. S4A).

**Fig. 3.**
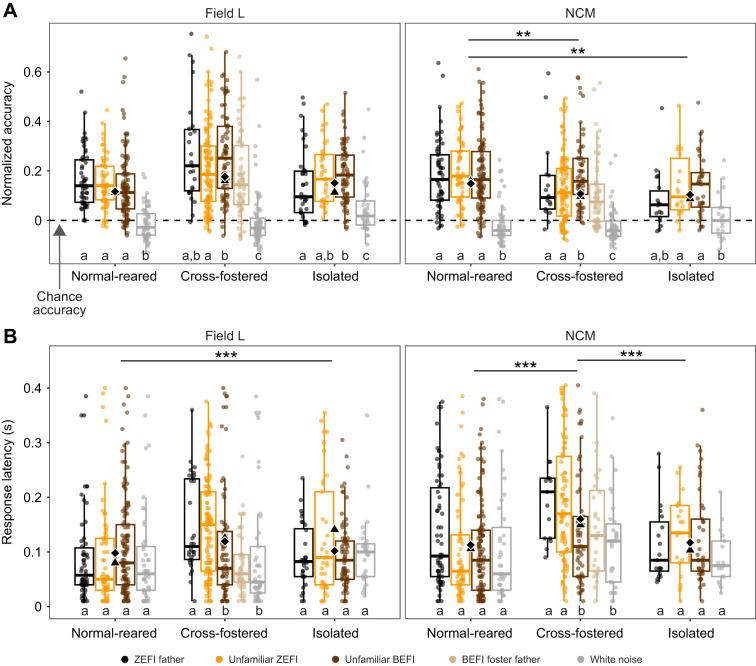
**Units from cross-fostered birds showed reduced firing pattern consistency and slower response latencies to conspecific song.** (A) Normalized *R*_corr_ accuracy classification scores as a function of brain region (panels), treatment and stimulus category (point color). The higher the score, the better the temporal firing pattern predicts the stimulus. A score of zero represents random classification, as shown by the horizontal dashed line. Units from cross-fostered birds showed higher accuracy values in response to uBEFI versus uZEFI songs in Field L (LMM with Tukey correction, *t*_706_=3.34, *P*=0.01) and NCM (LMM with Tukey correction, *t*_574_=2.94, *P*=0.02). Black diamonds show arithmetic means and black triangles show estimated marginal means for each treatment group. Letters below boxplots show contrasts within treatment groups with *P*>0.05. Lines above each plot show contrasts between treatment group means where **P<0.01. (B) Latency to respond to a given stimulus as a function of brain region (panels), treatment and stimulus category (point color). Cross-fostering resulted in slower latencies when responding to unfamiliar songs of the foster species. Each point represents a unit-stimulus combination. Each stimulus was presented to each unit (see sample sizes in Table 1) 15–20 times. Black diamonds show arithmetic means and black triangles show estimated marginal means for each treatment group. For both A and B, letters below boxplots show contrasts within treatment groups with *P*>0.05. Lines above each plot show contrasts between treatment group means where ***P<0.001.

Temporal coding consistency, as measured by normalized *R*_corr_ classification accuracy, was reduced by nearly half in units from cross-fostered and isolated birds as compared with those in normal-reared birds, again in NCM but not Field L (normal NCM mean=0.16±0.02, cross-fostered NCM mean=0.10±0.02; isolated NCM mean=0.09±0.02, contrast normal-reared versus cross-fostered estimate=0.06±0.02, *t*=2.91, *P*=0.01; contrast normal-reared versus isolated estimate=0.07±0.02, *t*=3.19, *P*<0.01; Fig. 3A). These differences in accuracy in NCM do not appear to be solely the result of higher firing rates in cross-fostered and isolated birds, given that firing rate differences were present in both regions.

We also found strong interactions between response latency, brain region and social rearing environment. In Field L, response latency for units from isolated birds (mean=0.14±0.02 ms) was 1.8× slower compared with units in normal-reared birds (mean=0.08±0.01 ms, estimate=−0.57±0.17, *t*=−4.91, *P*<0.0001). In NCM, response latency for units from cross-fostered birds (mean=0.15±0.01 ms) was 1.4× slower than in units from normal-reared birds (mean =0.11±0.01 ms, estimate =−0.35±0.09, *t*=−4.01, *P*<0.001; Fig. 3B).

Therefore, for prediction 1, an altered early social experience produced a clear disruption of ordinary neuronal response properties in the higher-order region NCM, including baseline firing rates, song responsiveness, song selectivity and temporal coding. In Field L, altered early social experience only affected firing rates.

### Prediction 2: heterospecific experience during the nestling phase reduces responsiveness, selectivity and firing consistency to conspecific song

After finding general electrophysiological differences across treatments, we looked for differences in how auditory neurons from birds in each treatment group responded to species song types. We saw overall that units from cross-fostered birds were less responsive and less selective for conspecific songs. Across all treatments, single units had higher *z*-scores in response to songs over white noise (Fig. 2B), as well as differences in responsiveness to zebra finch and Bengalese finch songs, including fathers' songs. There was no difference in *z*-scores of units from normal-reared birds to unfamiliar songs of the two species, but in both regions, units from isolated birds responded more strongly to unfamiliar zebra finch songs (uZEFI) over unfamiliar Bengalese finch songs (uBEFI; Field L estimate=−0.49±0.12, *t*=−4.15, *P*<0.001; NCM estimate=−0.39±0.15, *t*=−2.67, *P*=0.04). Conversely, NCM units from cross-fostered birds were 55% more responsive to uBEFI over uZEFI (estimate=0.44±0.09, *t*=4.96, *P*<0.0001; Fig. 2B). This pattern was not seen in Field L. We also saw that the proportion of NCM units from cross-fostered birds responsive to uZEFI was less than the proportion responsive to uBEFI (0.61 vs 0.81; Fig. S3B).

**Fig. 4.**
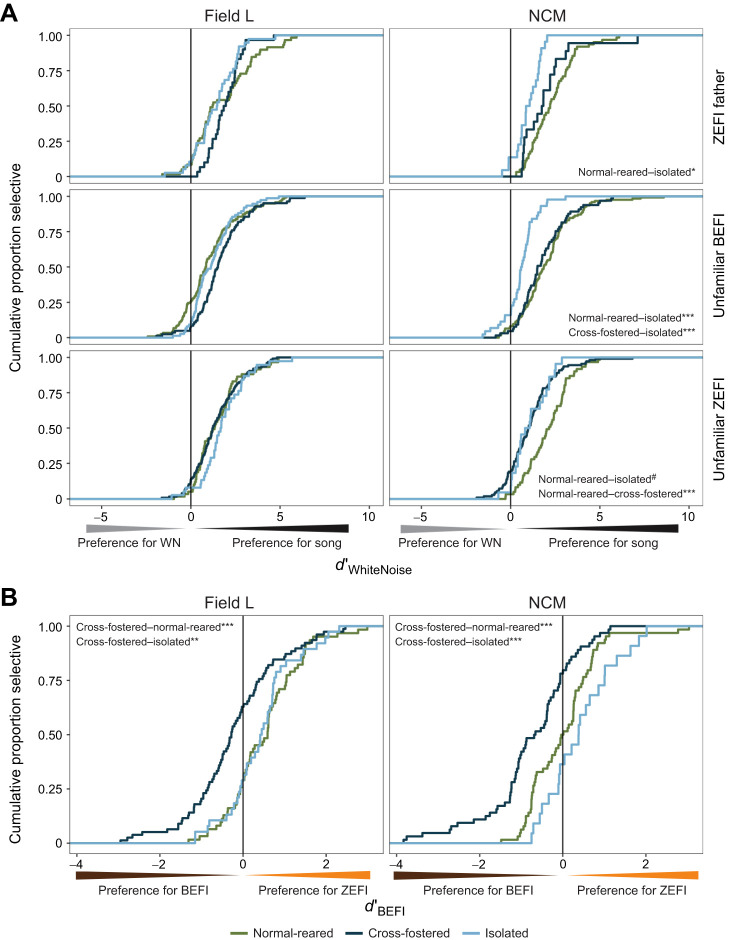
**Although units from isolated birds are less selective than units from normal-reared birds overall, units from cross-fostered birds are specifically less selective to zebra finch songs.** (A) Cumulative distribution functions on *d*′_WhiteNoise_ broken down by brain region (columns) and stimulus (rows). A reduction in selectivity of units from cross-fostered birds for uZEFI songs is evident in NCM (Levene's test with Tukey correction, difference=−1.16, *P*<0.0001), but not Field L. (B) Cumulative distribution functions on *d*′_BEFI_ show reduced selectivity for uZEFI compared with uZEFI in units from cross-fostered birds in both brain regions (Levene's tests with Tukey correction, Field L difference=−0.73, *P*<0.001; NCM difference=−0.78, *P*<0.0001). For treatment group pairwise comparisons, ***P<0.001, **P<0.01, *P<0.05, ^#^*P<*0.1.

We saw a similar pattern across treatments with stimulus selectivity (*d*′_WhiteNoise_) in NCM, where units from cross-fostered birds were less selective for uZEFI than uBEFI (estimate=0.65±0.13, *t*=4.89, *P*<0.0001; Fig. S4A) in contrast to the conspecific preference seen in units from normal-reared birds. Cumulative distribution analysis illustrates that this pattern was specifically due to a reduction in selectivity for uZEFI, rather than an increase in selectivity for uBEFI, relative to units from normal-reared birds (compare normal-reared and cross-fostered curves in right middle panel with those in the right bottom panel of Fig. 4A), rather than an increase in selectivity for uBEFI over white noise. To compare selectivity for species-specific songs directly, we also calculated *d*′_BEFI_, a measure of the neural selectivity for zebra finch songs over Bengalese finch songs. Units from cross-fostered birds showed greatly reduced selectivity for uZEFI directly over uBEFI in both regions (Field L cross-fostered–normal-reared=−0.73, *P*<0.001; NCM cross-fostered–normal-reared=−0.78, *P*<0.0001; Fig. 4B). Units from cross-fostered and isolated birds were less selective for fewer total songs relative to units from normal-reared birds in NCM (Fig. S4B).

Along with higher relative responsiveness and selectivity for Bengalese songs in units from cross-fostered birds, these units also showed higher consistency of firing patterns in response to Bengalese finch songs. Units from cross-fostered birds scored 6% higher classification accuracy values for uBEFI stimuli compared with uZEFI stimuli in Field L (*t*=3.34, *P*<0.01) and 5% higher in NCM (*t*=2.94, *P*=0.02; Fig. 3A), whereas the other two treatments showed no differences between those stimulus categories. This indicates that firing patterns better predict heterospecific songs only in cross-fostered birds.

Regarding response time, units from normal-reared and isolated birds showed no significant differences in latency to respond to any stimulus categories in either region. However, units from cross-fostered birds responded fastest to uBEFI and white noise, and slowest to zebra finch songs, in some cases twice as slow (NCM father ZEFI – white noise estimate=0.66±0.21, *Z* ratio (*z*-score of group mean contast) =3.21, *P*=0.01; Fig. 3B). This pattern opposes the (non-significant) trend in normal units, which respond fastest to uZEFI songs. Notably, in both regions, cross-fostered unit latencies did not differ from normal-reared unit latencies for uBEFI and white noise stimuli (all *P*>0.05), but units from cross-fostered birds were much slower to respond to zebra finch stimuli (Field L father ZEFI estimate=−0.77± 0.27; *Z* ratio=−2.88, *P*=0.01; Field L uZEFI estimate=−0.60±0.24; *Z* ratio=−2.48, *P*=0.03; NCM Father ZEFI estimate=−0.48±0.19; *Z* ratio=−2.59, *P*=0.03; NCM uZEFI estimate=−0.60±0.14; *Z* ratio=−4.40, *P*=<0.0001; Fig. 3B).

Therefore, for prediction 2, early experience with a heterospecific social environment biased auditory neuron responsiveness, selectivity and temporal firing patterns away from conspecific songs and toward heterospecific songs.

### Prediction 3: auditory forebrain responses to familiar versus unfamiliar stimuli do not differ

We compared responses of units from normal-reared birds to subjects' father's songs (familiar stimuli) versus uZEFI. Overall, there was generally no difference in responses to these two song types. We did see, however, that stimulus-evoked firing rates in NCM were higher to uZEFI (estimate=−0.08±0.02, *Z* ratio=−3.48, *P*<0.01; Fig. S3A) and that *z*-scores in Field L were higher to father's songs (estimate=0.34±0.11, *t*=3.10, *P*=0.01; Fig. 2B). Given the inconsistency of which stimulus evoked the stronger response in these two findings, and the lack of any differences across other metrics, our results suggest that zebra finches do not memorize individual song exemplars until the sensitive period. We did not compare responses to familiar and unfamiliar in units from cross-fostered birds because only one male Bengalese finch's song was used as a familiar stimulus.

### Behavioral attentiveness to conspecific song may be reduced in cross-fostered and isolated birds

**Fig. 5.**
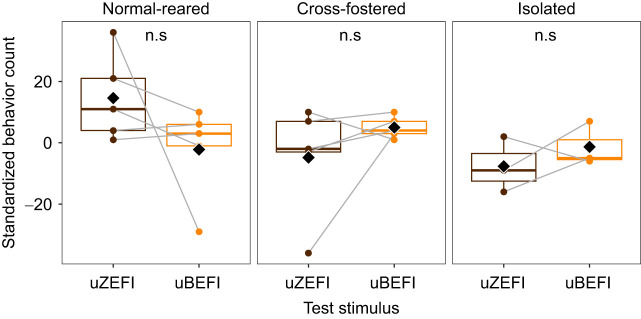
**Behavioral responses of fledgling zebra finches to uZEFI and uBEFI songs.** Standardized behavior count is the number of behaviors observed during 1 min of stimulus playback minus the number of behaviors during the 1 min of silence prior to playback. Behaviors include head turns, head tilts, neck stretches and gaping. Panels separate treatment groups. Gray lines connect trials with the same individual. Paired Wilcoxon signed-rank tests showed no significant differences in song responses in any treatment group.

With our modest sample for behavioral responses (*N*=5 normal-reared, 5 cross-fostered, 3 isolated birds), we did not detect significant differences in standardized behavioral scores in response to uZEFI versus uBEFI songs in any treatment group (normal-reared *V*=3, *P*=0.3; cross-fostered *V*=12, *P*=0.3; isolated *V*=5, *P*=0.5). Qualitatively, however, although birds across all treatments responded similarly to uBEFI, it appears that birds with less experience with zebra finch songs were less attentive to uZEFI (i.e. they performed fewer searching behaviors; Fig. 5).

## DISCUSSION

Our study provides evidence that songbirds are sensitive to species song categories prior to the sensitive period of vocal song learning. Our findings demonstrate that neural response properties in the auditory forebrain are governed by exposure to conspecific song and interaction during the early postnatal period. Cross-fostered birds showed neural and behavioral responses to zebra finch and Bengalese finch song that were drastically unlike those in normal-reared birds. Given that normal-reared and cross-fostered birds were raised in the same room with the same auditory environment, this suggests that the strong predisposition for conspecific responsiveness and selectivity seen in songbirds may be overridden by social interactions alone (Mets and Brainard, 2018), and that this can take place already during the nestling phase. Thus, nestling experience is important for molding auditory templates before sensorimotor learning itself begins.

A deficit in socially guided experience with conspecific song led to changes in units from cross-fostered and isolated birds when compared with units from normal-reared birds. This clearly parallels changes measured in adults following rearing environment manipulation (Cousillas et al., 2006; Hauber et al., 2013; Moore and Woolley, 2019; Woolley et al., 2010; Yanagihara and Yazaki-Sugiyama, 2016). Yet, units from cross-fostered and isolated birds in our study did not exhibit all of the same changes across treatments. Both groups were similar in that they showed an increase in firing rates relative to units from normal-reared birds, suggesting an experience-dependent alteration of baseline excitatory/inhibitory balance that could affect neural plasticity and learning. Both cross-fostered and isolated birds, but not normal-reared birds, also exhibited a population of ‘atypical broad’ cells, auditory neurons that were both symmetrical and long-duration in peak-to-peak duration waveform, suggestive of delayed developmental maturation. The circuit connectivity and membrane/ion channel properties that are sensitive to social and auditory experiences at the youngest rearing stages now become active areas of interest. A striking difference across treatment groups, however, was that units from cross-fostered birds showed a unique pattern of stimulus-specific reductions in responsiveness and selectivity only to conspecific songs. This is consistent with the idea that typical development is guided not simply by hearing adult conspecific songs, as cross-fostered birds still heard conspecific songs, but specifically by engaging with these sounds in a relevant social (parenting) context.

We did not see any consistent differences in the responses of units from normal-reared birds to familiar and unfamiliar stimuli, consistent with the prior evidence that nestlings do not yet memorize individual song stimuli (Braaten, 2010; but see Colombelli-Négrel et al., 2014; Eales, 1985; Roper and Zann, 2006). Nevertheless, embryo and nestling songbirds do show evidence of some level of perceptual learning, including incubation call imitation, physiological stimulus habituation (Colombelli-Négrel et al., 2012), stimulus-specific adaptation in NCM (Schroeder and Remage-Healey, 2021), and learning of subspecies song categories through experience (Schroeder and Podos, 2023). Thus, we do not rule out the possibility that experimentation focused on a comparison of responses to familiar and unfamiliar stimuli might reveal learning in zebra finches younger than 25 days. Additionally, the lower responsiveness and selectivity for conspecific songs seen in auditory neurons from cross-fostered birds in our study might underlie a learned preference for heterospecific songs, or at least an experience-dependent broadening of the range of acceptable tutor models.

We saw experience-dependent changes in auditory responses in both Field L and NCM. Notably, however, we saw that differences in auditory responsiveness, selectivity and response latency to zebra finch versus Bengalese finch songs after cross-fostering or isolation were more prevalent in the secondary region NCM than in the primary thalamorecipient Field L. There is conflicting evidence that conspecific selectivity in Field L is affected by experience (e.g. Araki et al., 2016; Moore and Woolley, 2019). In contrast, NCM is consistently altered by experience across studies (e.g. Kudo et al., 2020; Woolley and Doupe, 2008; Yanagihara and Yazaki-Sugiyama, 2016), so the stronger effects we saw in our data in NCM are not surprising. Additionally, there is evidence that spectrotemporal tuning for conspecific songs arises late in the primary forebrain pathway, Field L subregion L3 (Moore and Woolley, 2019). In our study, we did not precisely map the subregion locations of our Field L recording sites, but our results suggest that we predominantly sampled from L1 and L2, where conspecific selectivity may not be as abundant.

Early postnatal experience-dependent reorganization of perceptive tuning is ubiquitous across taxa (Schreiner and Polley, 2014; Woolley, 2017). Early malleability is important for adapting to one's social environment – perhaps accounting for things such as cultural and genetic shifts in communication signals – and may have a large impact on subsequent social behaviors in adults. Our study demonstrates plasticity in the development of auditory processing beginning in the nestling phase of a songbird. We found that early auditory and social interaction alter neural responsiveness and selectivity for conspecific vocal signals, which also could lead to a disruption of normal social attentive behavior. Given that these changes occurred before learned vocal signals are produced in this species, our results here suggest that nestling experience already causes changes to the auditory forebrain that could affect tutor song and mate choice later in life.

## Supplementary Material

10.1242/jexbio.247956_sup1 Supplementary information
