# Supplementary material for: Social and auditory experience shapes forebrain responsiveness in zebra finches before the sensitive period of vocal learning
Source: J Exp Biol. 2024 Oct 25;227(21):jeb247956. doi: 10.1242/jeb.247956 (PMC11529884; doi:10.1242/jeb.247956)
Supplement: Supplementary information [file jexbio-227-247956-s1.pdf]

Supplementary Materials and Methods

Additional Neural Response Metrics

The stimulus-evoked firing rate is the number of spikes/s during the exact duration of each individual stimulus.

To assess whether units were selective to a similar number and type of stimuli across treatments we calculated a measure of redundancy, called Proportion Selective, that counted the number of zebra finch stimuli (out of 2) and the number of Bengalese finch stimuli (out of 2) that each unit responded to with $d'_{WhiteNoise} > |0.7|$ (threshold as in Mooney et al., 2001).

We used a custom pattern classifier to assess consistency of spike timing in response to the different stimulus types. Because not all stimuli were the same duration, for this analysis we examined spike trains of a consistent duration from stimulus onset (1.2 s, the length of the shortest stimulus that any bird heard). In short, for each unit, a single spike train response to one presentation of each test stimulus was randomly chosen as the templates (i.e. 5 templates). At this point the program had no prior knowledge as to which stimulus these spike trains were responding. Then, the templates were iteratively compared to all remaining spike trains from each presentation of each stimulus, and each comparison was given a correlation value to generate a confusion matrix comparing the templates to the remaining set of spike trains. This process, beginning with random template selection, was conducted 1000 times for each unit and stimulus, then the R_{corr} method (Caras et al., 2015) was used to calculate the proportion of cases where the stimulus presentation with the highest correlation to the template was from the same stimulus. See further details in (Vahaba et al., 2017). R_{corr} accuracy ranges from 0 to 1, with higher values indicating that the spike timing patterns better predict the stimulus. If the classifier were randomly assigning stimuli to spike trains, the accuracy would be a value of 1/S, where S is the number of stimuli heard by each unit. The values reported in this manuscript are normalized to account for different numbers of stimuli heard by some units, therefore normalized R_{corr} accuracy is random if 0.

The latency to respond to each stimulus was calculated as in Ono et al. (Ono et al., 2016). We created 5 ms-binned peri-stimulus time histograms with boxcar smoothing. The latency was determined as the time bin after stimulus onset where the firing rate exceeded the

Journal of Experimental Biology • Supplementary information

mean baseline firing rate (100 ms prior to stimulus onset) by 3 standard deviations. If the unit did not exceed this threshold within 400 ms, it was excluded from further analysis.

Additional Statistical Analyses

We used Pearson correlations to look for a relationship between behavioral and electrophysiological responses to unfamiliar songs of each species. We chose z-score and $d'_{WhiteNoise}$ as the electrophysiology measures for comparison. For NCM units only, we calculated the average for each of these two metrics across units within a bird in response to unfamiliar zebra finch and unfamiliar Bengalese finch songs, thus there were two data points per bird per metric.

For the redundancy metric (described above), we performed a cumulative distribution function analysis (CDF) and used a Levene's test to analyze the variance.

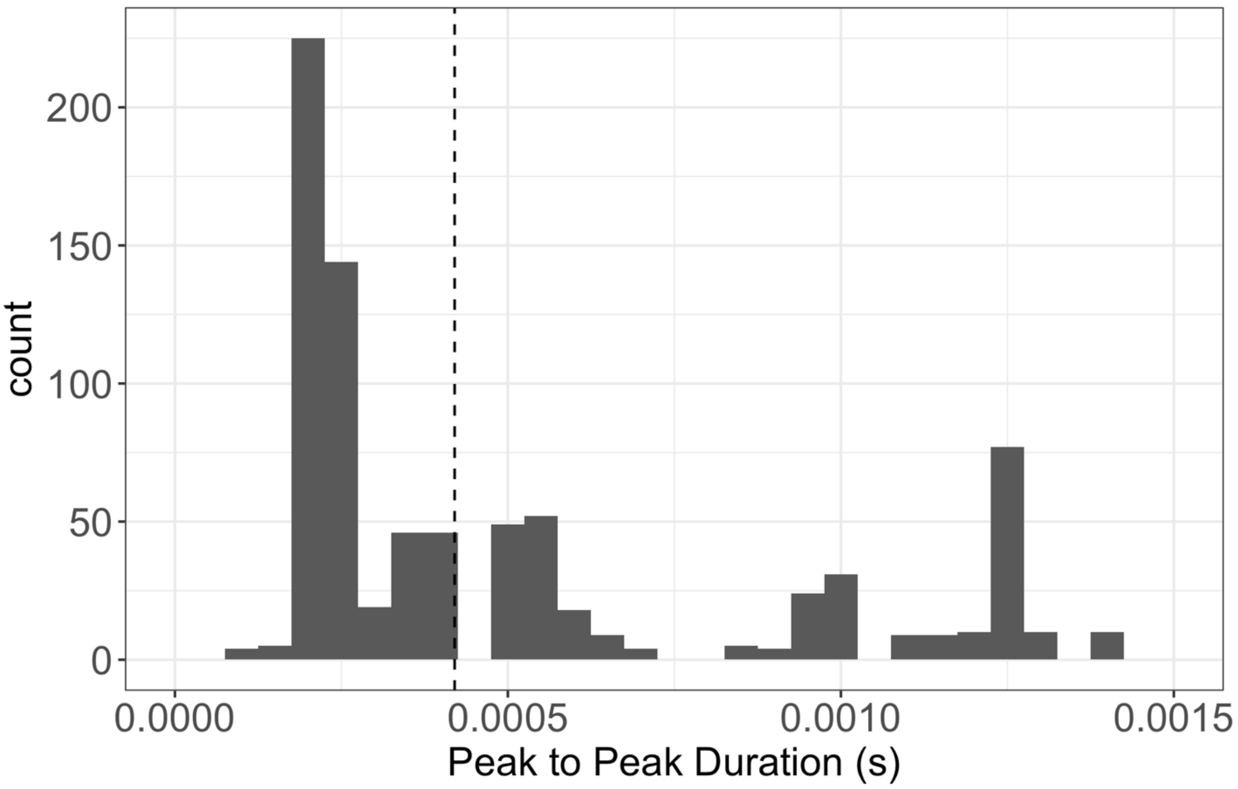

Fig. S1. Histogram of Field L single units. Dashed line represents the cutoff between NS and BS units, at 0.0004 ms.

Journal of Experimental Biology • Supplementary information

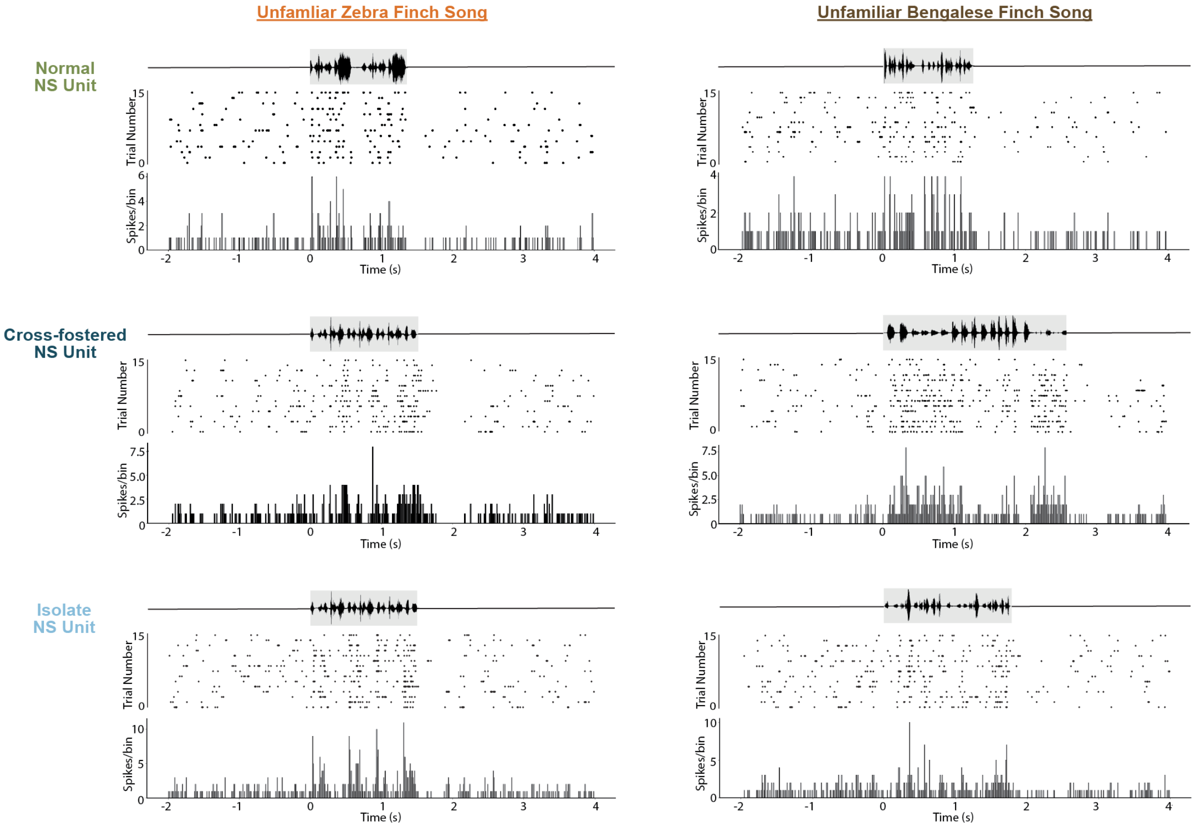

Fig. S2. Zebra finch and Bengalese finch song stimulus waveforms (top), raster plots (middle), and peristimulus time histograms (bottom) showing the activity of exemplar single NS units from NCM of a normal, cross-fostered, and isolate bird.

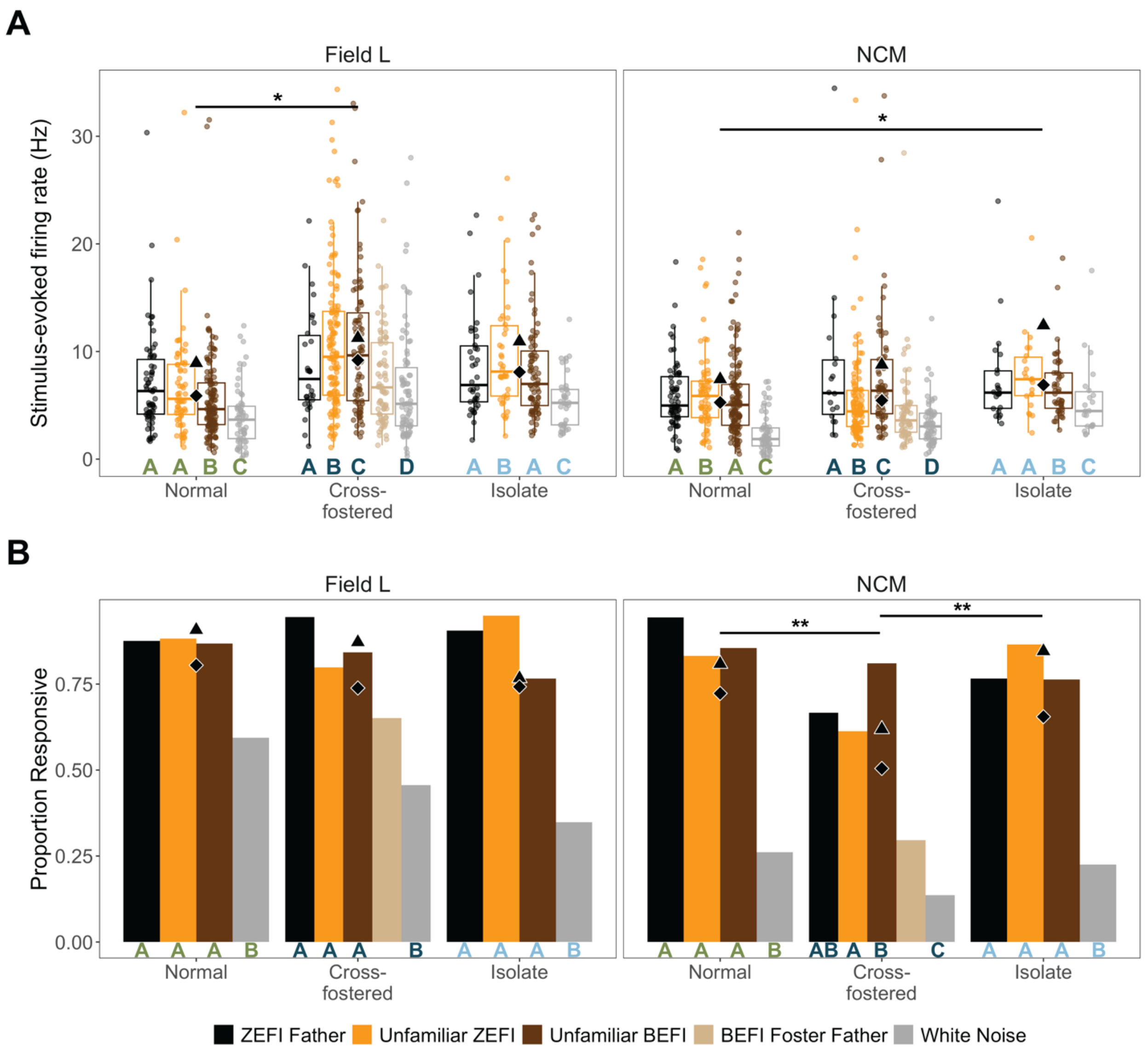

Fig. S3. A. Stimulus-evoked firing rates as a function of brain region (panels), treatment, and stimulus category (point color). The Foster Father stimulus is plotted here for visual comparison but was not included in statistical analyses (see discussion in Methods). Similar to the increase in baseline firing rates, stimulus-evoked firing rates were higher in units from isolate birds compared to units from normal birds in NCM, and stimulus-evoked rates in units from cross-fostered birds were higher than normal birds in Field L and no different in NCM. There were no differences either in NCM or Field L in baseline or stimulus-evoked firing rates between units from isolate and cross-fostered birds in either region. **B.** The proportion of units that responded with firing rates significantly over baseline to each stimulus category (bar colors) as a function

Journal of Experimental Biology • Supplementary information

of brain region (panels) and treatment. The odds of an NCM unit responding significantly over baseline firing rate to a given stimulus was lower in units from cross-fostered and isolate birds than in units from normal birds. Note also that the proportion of units from cross-fostered birds in NCM responsive to uZEFI was less than the proportion responsive to uBEFI, while this was not the case for the other two treatments. The Foster Father stimuli is plotted here for visual comparison but was not included in statistical analyses (see discussion in Methods). The proportion was first calculated within each bird, then averaged across treatment. Black diamonds show arithmetic means and black triangles show estimated marginal means for each treatment group. Letters below boxplots and barplots show contrasts within treatment groups with $P > 0.05$. Lines above each plot show contrasts between treatment group means where $^{***}P < .001$; $^{**}P < .01$; $^{*}P < .05$.

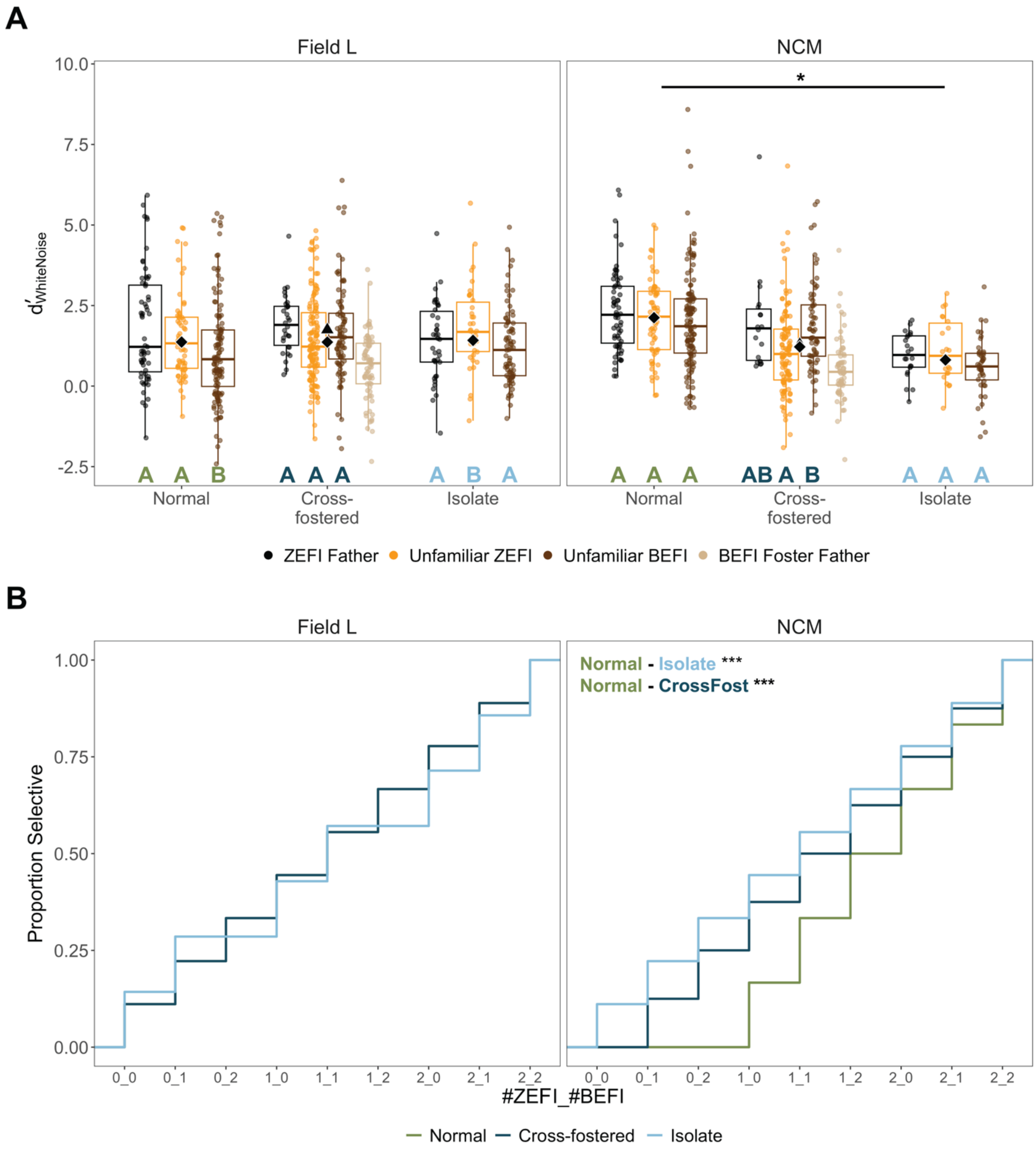

Fig. S4. A. d'$_{WhiteNoise}$ as a function of brain region (panels), treatment, and stimulus category (point color). Each point represents a unit-stimulus combination. **B.** Redundancy analysis of the total number of stimuli that each unit is selective for based on d'$_{WhiteNoise}$ > |0.7|. The x-axis shows categories for the NumberOfZEFISongs_NumberOfBEFISongs out of 2 ZEFI songs and 2 BEFI songs. All units were generally selective for most or all song stimuli and there were no differences between treatments in Field L. On the other hand, in NCM, single units from normal birds tended to be selective for more individual songs, particularly zebra finch songs, while units in isolate and cross-fostered birds are selective for fewer total songs.

Journal of Experimental Biology • Supplementary information

Supplementary Literature Cited

Caras, M. L., Sen, K., Rubel, E. W. and Brenowitz, E. A. (2015). Seasonal plasticity of precise spike timing in the avian auditory system. *J. Neurosci.* **35**, 3431–3445.

Mooney, R., Hoese, W. and Nowicki, S. (2001). Auditory representation of the vocal repertoire in a songbird with multiple song types. *Proc. Natl. Acad. Sci. U. S. A.* **98**, 12778–12783.

Ono, S., Okanoya, K. and Seki, Y. (2016). Hierarchical emergence of sequence sensitivity in the songbird auditory forebrain. *J. Comp. Physiol. A Neuroethol. Sensory, Neural, Behav. Physiol.* **202**, 163–183.

Vahaba, D. M., Macedo-Lima, M. and Remage-Healey, L. (2017). Sensory coding and sensitivity to local estrogens shift during critical period milestones in the auditory cortex of male songbirds. *eNeuro* **4**, ENEURO.0317-17.2017.
